# Prevention method preferences and factors influencing hypothetical choice among women in South Africa: a survey exploring opportunities for a multipurpose prevention technology implant

**DOI:** 10.3389/frph.2024.1368889

**Published:** 2024-06-25

**Authors:** Catherine E. Martin, Alison Kutywayo, Paballo Mataboge, Glory Chidumwa, Nqaba Mthimkhulu, Rutendo Bothma, Saiqa Mullick

**Affiliations:** Wits RHI, University of the Witwatersrand, Johannesburg, South Africa

**Keywords:** multipurpose prevention technology, MPT, PrEP, HIV prevention, contraception

## Abstract

**Introduction:**

South African women bear an intersecting burden of HIV, sexually transmitted infections (STIs) and unintended pregnancy. Multipurpose prevention technologies (MPTs) are a class of products that address multiple needs and have the potential to improve uptake and use of prevention products.

**Methods:**

Analysing survey data from 703 HIV-negative women 18–40 years in three provinces in South Africa, collected between July and November 2022, this study explores their preferences for prevention methods and factors influencing choice of hypothetical prevention methods, including MPTs. Descriptive statistics and multinomial regression analyses were conducted to determine prevention method preferences and factors associated with choosing a pill, injectable or MPT-implant type prevention method.

**Results:**

Most women wanted to prevent HIV, STIs and pregnancy. The most important factors when choosing a prevention product were whether it provided dual and long-term protection and if side effects were manageable. If choosing only one method, half of women would choose any MPT-implant and a quarter each would choose a pill or an injectable method, with method choices differing by population group.

**Discussion:**

Prevention method choices were influenced by sexual-behavioural factors and current and prior contraceptive method use. Providing a choice of prevention methods and a population specific approach to new method development and introduction with access to accurate information could enhance their ability to fill a gap in prevention needs.

## Introduction

Women in South Africa experience high rates of HIV, sexually transmitted infections (STIs) and unintended pregnancy (1–3). Addressing these coinciding epidemics through the integration of sexual and reproductive health (SRH) services has been a national focus (4, 5). Contraceptive methods available free of charge in public clinics in South Africa include condoms, oral and injectable contraceptives, intra-uterine devices and subdermal implants (6). Oral pre-exposure prophylaxis (PrEP) for the prevention of HIV was introduced to young women in South Africa in 2017 (7), with the dapivirine vaginal ring and long-acting injectable cabotegravir recently approved for use in country, but not yet available outside of implementation studies. Despite their availability, health system, interpersonal and product related barriers to uptake and effective use of contraceptives and oral PrEP remain, particularly among young people (8–13).

Multipurpose prevention technologies (MPTs) are a class of products that simultaneously prevent HIV, other STIs and/or unintended pregnancy (14). MPTs have the potential to improve uptake and use of prevention products by simplifying delivery and use, reducing stigma, providing discretion, and reducing user and health system burden (15–18). They may also offer additional protection among users who may otherwise have chosen a single-purpose product, in addition to being more cost-effective than single-purpose products (15–18). Currently, the condom is the only available MPT, although there are several MPTs in pre-clinical studies and clinical trials (14).

The development of new methods is complex and costly, and it is critical that in addition to expanding choice, investments are made in products most likely to be preferred and effectively used (19). Evidence from existing product introduction highlights that availability and efficacy do not always translate into uptake and effective use (19), and that they can be undermined by negative user experiences and misperceptions (20). In addition, health system considerations such as provider training requirements and community buy-in may impede new product introduction and use (21). Understanding end-user preferences is critical to inform considerations around which products should move forward in development; to ensure users are willing and able to use new products, and that they are appropriate for the contexts in which they will be implemented (17, 19, 21). This is even more important when effective single-purpose products are already available.

This study describes the prevention product choices and the factors associated with preferences for HIV prevention method types among women at risk of HIV in South Africa, with a particular focus on a hypothetical dual HIV prevention and contraceptive implant.

## Methods

This work was part of a study funded by the Bill and Melinda Gates Foundation. The main study was cross-sectional, mixed-methods, formative research among women, men and health care providers which aimed to investigate the potential uptake of a hypothetical MPT-implant among PrEP eligible clients in South Africa. The study was conducted between July and November 2022. This paper focuses on the findings from surveys conducted among female participants. Additional findings from the other components of this formative research have been published elsewhere (22–24).

### Study setting

The study was conducted in eight department of health primary care clinics and one key population clinic and their linked communities in three provinces in South Africa: Two clinics and a linked mobile clinic in peri-urban residential townships in Tshwane, Gauteng; two clinics and a linked mobile clinic in Mthatha in the rural Eastern Cape; and four clinics within two distinct rural communities in KwaZulu Natal. Recruitment of female sex workers (FSWs) was undertaken at a Wits RHI supported key populations clinic and linked mobile clinic in Tshwane. All study sites are in communities with a high burden of HIV, with antenatal prevalence rates ranging from 23% in Tshwane to 35% in Mthatha (3). All sites have been supported by Wits RHI to introduce oral PrEP through implementation science projects.

### Study population and recruitment

Participants were HIV negative females aged 18–40 years. Female clients accessing services at recruiting sites were eligible if they were 18–40 years old, eligible for PrEP (self-reported HIV-negative) and willing and able to consent to study participation. Recruitment was conducted by trained fieldworkers and supported by peer educators at the key population site. For all target populations, two approaches to recruitment were used: consecutive sampling of clients accessing SRH services at study sites, and snowball sampling through already recruited participants, to allow for participation of women accessing health services as well as those from the community. Participants recruited at study sites were invited to present to a designated community venue on a set time and day to participate in study activities. Recruitment flyers were given to willing participants to take home to enable the recruitment of eligible family members or friends through snowballing. Flyers contained a study contact number, allowing participants to reach the study team for further information. If interested in participating, they were invited to present to the designated community venue on a set day and time, at which point they were screened and enrolled.

### Study procedures

All participants were provided with a standard, interactive group information session of approximately 45 min, facilitated by a trained study team member. Information on new, existing and hypothetical HIV prevention and MPT methods, provided through a slide presentation, covered details of each potential method i.e., daily, monthly, and event-driven pill, two-monthly and six-monthly injectable, 1-year biodegradable, non-biodegradable and refillable MPT implant, mono-PrEP implant and 2-year biodegradable and non-biodegradable MPT implant. For each, information on product administration, conditions prevented, potential side effects, duration of prevention, anticipated availability and effectiveness (where known) was provided. This was followed by a question and answer session with each group to answer any questions related to the information provided.

A sub-set of 299 (42.5%) participants (99 (33.1%) AGYW, 160 (53.5%) women >24 years and 40 (13.4%) FSWs) participated in a workshop immediately following the information session. The aim of the workshops was to explore demand creation messaging and channels as well as preferences for different attributes of a hypothetical MPT implant. There were four participants in the main study who completed a workshop, but for whom survey data was not included in this analysis as they did not complete the survey (*n* = 1) and were >40 years (*n* = 3). All FSWs participated in a workshop and recruitment for workshops among AGYW and women >24 years was conducted until data saturation was reached, after which participants completed only the information session. Workshops used participatory action research (PAR) methods and aimed to explore user preferences for prevention methods, perceptions of MPTs and to co-develop demand creation strategies for future MPTs. Workshops were approximately four hours, facilitated by two members of the study team and observed by three to four study team members. There were fourteen workshops in total, grouped according to population of interest: two among FSWs, six among AGYW and six among women >24 years. Each completed a set of activities which differed by population group. There was a median of 21 participants per workshop (ranging from 6 to 53). Mataboge et al. provide a full description of the workshop activities as well as the results of the PAR activities (22). In summary, this included an activity where participants voted for their favourite demand creation message for MPTs and an activity in which participants selected their most preferred, of three possible options, for each of eight identified attributes of a hypothetical MPT implant i.e., body placement, prevention characteristic (duration), side effects, service access point, removal options, replacement options, visibility and pain. FSWs participated in an activity which involved the identification and ranking of preferred characteristics of a hypothetical MPT. These activities were used to stimulate discussion and further enquiry, to gain a better understanding of participant choices and preferences.

Following workshops, participants completed a self-administered questionnaire on a handheld tablet, computer, or cell phone. For participants who did not participate in a workshop, the questionnaire was completed immediately following the information session. Questionnaire data were managed using REDCap electronic data capture tools hosted at the University of the Witwatersrand (25, 26). The questionnaire was in English and self-administered. Where assistance was requested, a study team member provided guidance in completing the survey.

### Measures

Survey data included demographics and self-reported sexual behaviour. Self-perceived risk of HIV, STIs, and pregnancy was categorised as either “no perceived risk”, or “some perceived risk” based on participant's own risk perception. Early sexual debut was defined as having first sexual intercourse at age ≤14 years. Transactional sex was defined as having a sexual relationship in the last three months in exchange for any of the following items: food, clothing, cosmetics, cell phone, items for children or family, transport or tickets, school or residence fees, cash or somewhere to stay.

Data were collected on the factors influencing hypothetical prevention method choice. Participants viewed a list of 15 factors identified to influence pregnancy, HIV and STI prevention method decision making, based on a previous MPT acceptability study (27) and were asked to rank these in order of importance to their prevention method choice from 1 to 15. These were not specific to MPTs, but about prevention methods more broadly. The most important factor was defined as the factor ranked number one by each participant.

Data on whether participants would consider the use of various HIV and pregnancy prevention options were also collected. Participants viewed a list of different prevention methods, aligned to those presented in the participant information session, and were asked for each if they would consider using it to protect themselves from getting HIV. Prevention method options were limited to injectable, oral and implant products to reflect the most widely used existing methods with which women in South Africa have experience. These were a once daily pill; monthly pill; two-monthly, gluteal, intra-muscular injectable (in keeping with provision of cabotegravir); six-monthly sub-cutaneous injectable (2 injections per dose) (in keeping with provision of lenacapavir); They were then asked if they would consider the following MPT-implant methods to prevent HIV and pregnancy: 1-year non-biodegradable; 1-year biodegradable; 1-year refillable; 2-year non-biodegradable; 2-year biodegradable. Participants were then asked to select from the methods previously listed, the method they would choose if they could only choose one. From these responses, participants were categorized as either preferring MPT PrEP if they chose any of the MPT-implants; pill PrEP if they chose the pill products; or injectable PrEP if they chose any of the injectable products. Those that would use none of the products were categorized as not choosing any form of PrEP. Prevention methods included in the questionnaire were restricted to those provided through oral, injectable and implant forms, as these are currently the most frequently used biomedical contraception and HIV prevention method forms, and the method forms with which the population had some existing knowledge and experience.

### Statistical analysis

A total of 703 females completed a study survey. Descriptive analyses of demographic and sexual behaviour characteristics, by population group of interest, were conducted. We determined the factors ranked most important when choosing a prevention product and which entity (HIV, STIs, or pregnancy) participants wanted to prevent. We determined the proportion of participants willing to consider each of the prevention methods presented, and their choice if choosing only one. We used multinomial logistic regression to assess the factors associated with choosing a pill, injectable or MPT-implant as a preferred prevention method among those who would consider any prevention product. The final regression model adjusted for age, study site and workshop participation *a priori*. Baseline factors with a *p*-value of 10% or less were included in the multivariable analysis and statistical significance was set at 5%. All statistical analyses were performed in STATA statistical software, version 15 (28).

## Results

Of the 703 women enrolled, 193 (27%) were recruited through snowballing and 508 (72%) through direct recruitment at study sites. Data on recruitment type was missing for two participants. Compared to AGYW (89/289, 31%) and women >24 years (99/374, 26%), FSWs were recruited less frequently through snowballing (5/40, 12.5%).

### Demographics

The demographic and sexual behavioural characteristics are presented in Table 1, stratified by population group. Most participants had completed secondary school, with completion of tertiary level education ranging from 38.0% (142/374) among women >24 years to 12.5% (5/40) among FSWs. Almost half of AGYW were students (46.0%, 133/289), whereas most women >24 years (65.0%, 243/374) were unemployed.

**Table 1:** Demographic and sexual behavioural characteristics of participants, by population group.

	AGYW 18–24 years *N* = 289 (41.1%)		Women >24 years *N* = 374 (53.2%)		Female sex workers *N* = 40 (5.7%)		Total *N* = 703 (100%)	
Age (years)								
Mean (SD)	20.9	(2.00)	30.7	(4.37)	31.7	(6.09)	26.7	(6.15)
Study site								
KZN	99	34.3%	149	39.8%	0	0.0%	248	35.3%
Gauteng	102	35.3%	104	27.8%	40	100.0%	246	35.0%
Eastern Cape	88	30.4%	121	32.4%	0	0.0%	209	29.7%
Sexual orientation								
Heterosexual	256	88.6%	358	95.7%	30	75.0%	644	91.6%
Bisexual/homosexual	28	9.7%	13	3.5%	7	17.5%	48	6.8%
Missing	5	1.7%	3	0.8%	3	7.5%	11	1.6%
Highest level of education completed								
Primary school or lower	12	4.2%	10	2.7%	4	10.0%	26	3.7%
Secondary school	210	72.7%	215	57.5%	28	70.0%	453	64.4%
College or university	60	20.8%	142	38.0%	5	12.5%	207	29.4%
Missing	7	2.4%	7	1.9%	3	7.5%	17	2.4%
Employment								
Student	133	46.0%	37	9.9%	2	5.0%	172	24.5%
Employed (full/part time/self)	26	9.0%	87	23.3%	20	50.0%	133	18.9%
Unemployed	124	42.9%	243	65.0%	17	42.5%	384	54.6%
Missing	6	2.1%	7	1.9%	1	2.5%	14	2.0%
Ever had sexual intercourse	257	88.9%	367	98.1%	40	100.0%	664	94.5%
Early sexual debut	8	2.8%	10	2.7%	2	5.0%	20	2.8%
Ever given birth	99	34.3%	307	82.1%	37	92.5%	443	63.0%
Relationship								
No sexual partner	83	28.7%	95	25.4%	10	25.0%	188	26.7%
Casual partners	73	25.3%	88	23.5%	11	27.5%	172	24.5%
Married or in a committed relationship	120	41.5%	173	46.3%	11	27.5%	304	43.2%
Other/unknown	13	4.5%	18	4.8%	8	20.0%	39	5.5%
Knowledge of partner's HIV status*								
Known	133	64.6%	167	59.9%	20	66.7%	320	62.1%
Unknown	73	35.4%	112	40.1%	10	33.3%	195	37.9%
More than one sexual partner in the last 3 months*	13	6.3%	24	8.6%	15	50.0%	52	10.1%
Condom use at last sex**	124	48.3%	191	52.0%	24	60.0%	339	51.0%
Transactional sex in the last 3 months	24	8.3%	55	14.7%	32	80.0%	111	15.8%
Trying to conceive	22	7.6%	38	10.2%	4	10.0%	64	9.1%
Current contraceptive use**								
None^†^	87	33.9%	96	26.2%	10	25.0%	193	29.0%
Condoms only	15	5.8%	50	13.6%	7	17.5%	72	10.8%
Implant	37	14.4%	40	10.9%	7	17.5%	84	12.7%
Injectable	89	34.6%	127	34.6%	7	17.5%	223	33.6%
IUD	1	0.4%	4	1.1%	0	0.0%	5	0.8%
Pill	20	7.8%	35	9.5%	8	20.0%	63	9.5%
Female or male sterilization	3	1.2%	5	1.4%	0	0.0%	8	1.2%
Unknown	5	2.0%	10	2.7%	1	2.5%	16	2.4%
Used a contraceptive implant before	52	18.0%	96	25.7%	13	32.5%	161	22.9%
Ever tested for HIV	268	92.7%	355	94.9%	38	95.0%	661	94.0%
Ever had an STI	57	19.7%	105	28.1%	14	35.0%	176	25.0%
Ever used PrEP before	118	40.8%	73	19.5%	34	85.0%	225	32.0%
Ever used PEP before	41	14.2%	63	16.8%	12	30.0%	116	16.5%
Some perceived risk of HIV	61	21.1%	124	33.2%	17	42.5%	202	28.7%
Some perceived risk of STI	92	31.8%	166	44.4%	22	55.0%	280	39.8%
Some perceived risk of pregnancy	84	29.1%	142	38.0%	15	37.5%	241	34.3%

* Among those with a partner (*n* = 515).

** Among those who had ever had sex (*n* = 664).

^†^ Includes those who reported not using contraceptives or using only abstinence, emergency contraception or traditional contraceptive methods (e.g., withdrawal).

### Sexual behaviour and perceived risk of HIV, STIs and pregnancy

Almost all participants reported having had sexual intercourse, with early sexual debut ranging from 2.7% (10/374) among women >24 years to 5.0% (2/40) among FSWs. Almost all (92.5%, 37/40) FSWs, 82.1% (307/374) of women >24 years and a third of AGYW (34.3%, 99/289) had given birth. Overall, 43.2% (304/703) were married or in committed relationships, 26.7% (188/703) had no sexual partner and 24.5% (172/703) had casual partners. The majority of participants with partners knew their partner's HIV status (62.1%, 320/515), and 10.1% (52/515) had more than one sexual partner in the last three months. Half of sexually active participants (51.0%, 339/664) used a condom at last sex. Transactional sex ranged from 8.3% (24/289) among AGYW to 80% (32/40) among FSWs. The injectable was the most commonly used contraceptive among AGYW (34.6%, 89/257) and among women >24 years (34.6%, 127/367); the oral pill was most commonly used among FSWs (20.0%, 8/40). The implant was used by 17.5% (7/40) of FSWs, 14.4% (37/257) of AGYW and 10.9% (40/367) of women >24 years. Approximately a quarter (22.9%, 161/703) had used a contraceptive implant before.

Almost all participants (94.0%, 661/703) had tested for HIV, and 25.0% (176/703) reported ever having an STI. A third (32.0%, 225/703) had ever used PrEP, ranging from 19.5% (73/374) among women >24 years, to 85% (34/40) among FSWs, with fewer (16.5%, 116/703) having used post exposure prophylaxis. Just under a third (28.7%, 202/703) perceived themselves to be at risk of HIV, ranging from 21.1% (61/289) among AGYW to 42.5% (17/40) among FSWs; over a third perceived themselves to be at risk of STIs (39.8%, 280/703) and pregnancy (34.3%, 241/703).

Compared to those who participated only in the information session, AGYW and women >24 years who participated in the information session and workshops were significantly older; they also differed significantly by study site, employment status, having had sex, having given birth, knowledge of their partner's HIV status, condom use, and perceived risk of STIs (Supplementary Table S1). All FSWs participated in an information session and workshop.

### Prevention product preferences

The factors reported as the most important in decision making around prevention product choice are presented in Table 2, together with prevention method preferences. Dual protection was reported by 20.2% (142/703) as the most important factor when choosing a prevention product, followed by whether side effects were manageable (18.9%, 133/703) and whether it offers long term protection (12.2%, 86/703). These factors differed significantly by population group (*p* = 0.016).

**Table 2:** Hypothetical prevention product choices among participants, by population group.

	AGYW 18–24 years *N* = 289 (41.1%)		Women >24 years *N* = 374 (53.2%)		Female sex workers *N* = 40 (5.7%)		Total *N* = 703 (100%)		*p*-value
Most important factor in choice of prevention product									
Provides dual protection	65	22.5%	65	17.4%	12	30.0%	142	20.2%	0.016
Side effects are manageable	33	11.4%	93	24.9%	7	17.5%	133	18.9%	
Offers long term protection	39	13.5%	42	11.2%	5	12.5%	86	12.2%	
Size of product	16	5.5%	35	9.4%	1	2.5%	52	7.4%	
Convenient to use	19	6.6%	20	5.3%	4	10.0%	43	6.1%	
Available for use	17	5.9%	17	4.5%	1	2.5%	35	5.0%	
Dissolvable	12	4.2%	18	4.8%	3	7.5%	33	4.7%	
Flexible/suits my lifestyle	20	6.9%	9	2.4%	2	5.0%	31	4.4%	
Administration by a provider required	13	4.5%	13	3.5%	1	2.5%	27	3.8%	
Effectiveness	11	3.8%	12	3.2%	1	2.5%	24	3.4%	
Method of use	10	3.5%	11	2.9%	1	2.5%	22	3.1%	
Low user burden	6	2.1%	8	2.1%	1	2.5%	15	2.1%	
Avoidance of painful removal and scarring	9	3.1%	4	1.1%	0	0.0%	13	1.8%	
Discreet	7	2.4%	4	1.1%	1	2.5%	12	1.7%	
Frequency of dosing	4	1.4%	3	0.8%	0	0.0%	7	1.0%	
Missing	8	2.8%	20	5.3%	0	0.0%	28	4.0%	
What you would like to prevent									
Only one (HIV/STI/pregnancy)	11	3.8%	33	8.8%	2	5.0%	46	6.5%	0.082
HIV & Pregnancy	61	21.1%	59	15.8%	5	12.5%	125	17.8%	
HIV & STI	14	4.8%	26	7.0%	2	5.0%	42	6.0%	
HIV, pregnancy & STI	202	69.9%	251	67.1%	30	75.0%	483	68.7%	
Unknown	1	0.3%	5	1.3%	1	2.5%	7	1.0%	
Would consider using for the prevention of HIV									
Once daily pill	100	34.6%	173	46.3%	23	57.5%	296	42.1%	0.001
Monthly pill	178	61.6%	244	65.2%	30	75.0%	452	64.3%	0.216
Two monthly injectable	137	47.4%	199	53.2%	21	52.5%	357	50.8%	0.325
Six monthly injectable	138	47.8%	204	54.5%	20	50.0%	362	51.5%	0.218
Would consider using for the prevention of HIV and pregnancy									
1-year MPT non-biodegradable implant	142	49.1%	227	60.7%	27	67.5%	396	56.3%	0.004
1-year MPT biodegradable implant	144	49.8%	184	49.2%	23	57.5%	351	49.9%	0.607
1-year refillable MPT implant	103	35.6%	182	48.7%	27	67.5%	312	44.4%	<0.001
2-year non-biodegradable MPT implant	126	43.6%	205	54.8%	17	42.5%	348	49.5%	0.011
2-year biodegradable MPT implant	144	49.8%	176	47.1%	22	55.0%	342	48.6%	0.553
Would consider any 1- or 2-year MPT implant									
	246	85.1%	333	89.0%	38	95.0%	617	87.8%	0.111
Choice if only one prevention method available									
Once daily pill	26	9.0%	19	5.1%	1	2.5%	46	6.5%	0.001
Monthly pill	55	19.0%	53	14.2%	3	7.5%	111	15.8%	
Two monthly injectable	19	6.6%	36	9.6%	1	2.5%	56	8.0%	
Six monthly injectable	46	15.9%	75	20.1%	2	5.0%	123	17.5%	
1-year MPT non-biodegradable implant	16	5.5%	40	10.7%	2	5.0%	58	8.3%	
1-year MPT biodegradable implant	28	9.7%	37	9.9%	9	22.5%	74	10.5%	
1-year refillable MPT implant	16	5.5%	21	5.6%	7	17.5%	44	6.3%	
2-year non-biodegradable MPT implant	30	10.4%	32	8.6%	6	15.0%	68	9.7%	
2-year biodegradable MPT implant	46	15.9%	53	14.2%	9	22.5%	108	15.4%	
None	6	2.1%	3	0.8%	0	0.0%	9	1.3%	
Missing	1	0.3%	5	1.3%	0	0.0%	6	0.9%	

Most participants (68.7%, 483/703) wanted to prevent HIV, pregnancy and STIs. Almost half would consider using each of the prevention method options presented, ranging from 42.1% (296/703) who would consider the daily oral pill to 64.3% (452/703) who would consider the monthly pill. Across all participants, the monthly pill was most frequently considered, ranging from 61.6% (178/289) among AGYW to 75% (30/40) among FSWs. Methods noted to differ significantly across population groups were the daily pill (*p* = 0.001), the 1 and 2-year non-biodegradable (*p* = 0.004 and *p* = 0.011) and the refillable MPT-implant (*p* < 0.001). Methods noted to differ significantly between those who participated in the information session, and those who participated in the information session and workshop were the 6-monthly injectable (*p* = 0.010), the 1-year non-biodegradable (*p* < 0.001) and the 1-year refillable MPT-implant (*p* = 0.028) (Supplementary Table S2).

When choosing only one prevention method, the 6-monthly injectable was chosen most frequently (17.5%, 123/703), followed by the monthly oral pill (15.8%, 111/703) and the 2-year biodegradable MPT-implant (15.4%, 108/703). The methods chosen least frequently were the 1-year refillable MPT-implant (6.3%, 44/703), the daily oral pill (6.5%, 46/703), and the two monthly injectable (8.0%, 56/703). Overall, any MPT-implant was chosen by 50.1% (352/703) of participants. Choices differed significantly by population group (*p* < 0.001), with AGYW preferring the monthly oral pill (19.0%, 55/289), women >24 years the 6-monthly injectable (20.1%, 75/374) and FSWs the 1-year and 2-year biodegradable MPT-implants (each 22.5%, 9/40). Choices differed significantly between those who participated in an information session, and those who participated in an information session and workshop (*p* < 0.001) (Supplementary Table S2). Those who participated only in the information session preferred a monthly pill (19.3%, 78/404), whilst those who participated in the information session and workshop preferred the 2-year biodegradable MPT-implant (15.7%, 47/299).

### Factors associated with prevention product choices

The MPT-implant group was used as the reference category in the multinomial regression analysis (Table 3). All models adjusted for age, study site and workshop participation. Sex workers were less likely to choose an injectable compared to MPT-implant (RRR: 0.21, 95% CI: 0.05–0.78) and women who had sex before were less likely to choose an oral pill compared to an MPT-implant (RRR: 0.32, 95% CI: 0.12–0.89). Compared to those that were single, women who had casual partners were also less likely to choose an oral pill over an MPT-implant (RRR: 0.54, 95% CI: 0.30–0.97). Women who reported using condoms at last sex and who were trying to conceive were more likely to choose an oral pill over an MPT-implant (RRR: 1.70, 95% CI: 1.10–2.64: and RRR: 2.14, 95% CI: 1.03–4.48, respectively). Compared to those not using a contraceptive method, women who were currently using an implant (RRR: 0.38, 95% CI: 0.17–0.88) or an injectable contraceptive (RRR: 0.55, 95% CI: 0.31–0.96) were less likely to choose an oral pill over an MPT-implant. Women who had ever used a contraceptive implant were less likely to choose an injectable compared to MPT-implant (RRR: 0.54, 95% CI: 0.31–0.97). Relative to those who participated in the information session only, those who participated in both the information session and workshop were less likely to choose either a pill or an injectable compared to an MPT-implant (RRR: 0.48, 95% CI: 0.31–0.76 and RRR: 0.46, 95% CI: 0.31–0.70).

**Table 3:** Fully adjusted analyses for factors associated with hypothetical prevention method choice among females who would consider PrEP (*N* = 688).

	Pill compared to MPT-implant		Injectable compared to MPT-implant	
	RRR (95% CI)	*p*-value	RRR (95% CI)	*p*-value
Age	0.98 (0.93; 1.02)	0.272	1.03 (0.99; 1.07)	0.168
Enrolment phase				
Participated in information session only	Reference		Reference	
Participated in information session and workshop	0.48 (0.31; 0.76)	**0.001**	0.46 (0.31; 0.70)	**<0.001**
Study site				
KZN	Reference		Reference	
Gauteng	1.06 (0.62; 1.82)	0.829	1.35 (0.81; 2.27)	0.250
Eastern Cape	0.78 (0.46; 1.33)	0.364	1.09 (0.67; 1.78)	0.736
Sex worker				
Yes	0.37 (0.11; 1.24)	0.107	0.21 (0.05; 0.78)	**0.021**
Ever given birth				
Yes	1.10 (0.65; 1.88)	0.721	1.15 (0.69; 1.92)	0.598
Ever had sex				
Yes	0.32 (0.12; 0.89)	**0.029**	0.39 (0.13; 1.16)	0.090
Relationship				
Single	Reference		Reference	
Casual partners	0.54 (0.30; 0.97)	**0.038**	0.62 (0.35; 1.09)	0.099
Married or committed relationship	0.88 (0.53; 1.47)	0.631	1.16 (0.71; 1.90)	0.554
Other/unknown	0.78 (0.31; 1.94)	0.592	0.55 (0.20; 1.48)	0.236
Condom use at last sex				
Yes	1.70 (1.10; 2.64)	**0.017**	1.24 (0.83; 1.87)	0.295
Trying to conceive				
Yes	2.14 (1.03; 4.48)	**0.043**	1.78 (0.84; 3.77)	0.134
Current contraceptive method				
None	Reference		Reference	
Condoms	0.53 (0.24; 1.15)	0.110	0.68 (0.33; 1.42)	0.304
Implant	0.38 (0.17; 0.88)	**0.023**	0.60 (0.25; 1.39)	0.231
Injectable	0.55 (0.31; 0.96)	**0.037**	1.10 (0.65; 1.88)	0.717
Pill	1.40 (0.67; 2.93)	0.370	0.93 (0.42; 2.06)	0.859
Other/unknown	0.22 (0.06; 0.80)	0.021	0.32 (0.10; 1.05)	0.060
Ever used a contraceptive implant				
Yes	0.98 (0.55; 1.76)	0.959	0.54 (0.31; 0.97)	**0.038**
Ever tested for HIV				
Yes	0.60 (0.25; 1.46)	0.262	0.73 (0.30; 1.80)	0.500
Used PrEP before				
Yes	0.98 (0.60; 1.60)	0.931	1.03 (0.65; 1.63)	0.892
Side effects are manageable				
Yes	0.84 (0.49; 1.44)	0.528	1.25 (0.78; 2.02)	0.349
Provides dual protection				
Yes	0.71 (0.42; 1.20)	0.199	0.86 (0.53; 1.42)	0.564

## Discussion

MPTs present an opportunity to address multiple prevention needs among women. The results from this study are consistent with others, which provide a strong indication that women want to prevent pregnancy, HIV and STIs, and would prefer an MPT over single-purpose products (17, 18, 27, 29–33). The prevention methods most preferred by women in this study were a 6-monthly sub-cutaneous injection, followed by a monthly pill and a 2-year biodegradable implant, with half of women choosing any MPT-implant method and a quarter each choosing a pill or an injectable method. Women who had casual partners, who were not using condoms, who were not trying to conceive and who were engaged in sex work were significantly more likely to consider an MPT-implant as a prevention method.

Our findings confirm that, when looking at products that require consistent use, there is a preference among women for prevention methods that are long-acting, that provide dual protection, and have minimal side effects (16–18, 29, 30, 32, 34–36). Whilst having minimal side effects was noted to be of high importance to women in this study, and has been raised as a concern among young women in prior research (37), women may be willing to accept some product side effects if they are adequately counselled and supported to manage them (38). In contrast to studies conducted among men and women in the region (30, 31, 36, 39, 40), method efficacy was not reported to be one of the most important factors influencing choice in this study. This highlights the important role that other product attributes may play among women and reiterates the notion that product efficacy alone will not necessarily translate to its acceptability and use (40). Further evaluation of end-users understanding of efficacy may also be needed. There may be an assumption among end-users that longer-acting products have higher efficacy (34), or that products would only be made available if they met certain efficacy requirements.

Our findings support others which suggest that prior use of a method type may increase its acceptability and use (33). The TRIO study, a randomised cross over study of placebo MPTs found that prior use of a product was associated with a preference for that product at baseline (41) and that product ratings increased with use, indicating that the experience of using a product may increase its acceptability (16). A discrete choice experiment among women in South Africa and Kenya also found product experience to influence preferences for prevention method attributes (30). Formative studies exploring acceptability of MPT implants in clinical development have also noted the influence of prior implant use on user acceptability (42). It is notable that participation in this study's workshop was associated with choosing an MPT-implant. This may be due to the inherent differences in the characteristics of the population that participated in the workshops. However, may indicate that providing comprehensive information on new products as well as participatory activities reflecting on new products could influence choice, and may improve acceptability. As has been noted by others, community experiences and efforts to support new product users are likely to influence demand and adoption of new products (20, 30).

The findings from this study further highlight that daily pills are not a preferred prevention method (16, 30, 31), and reiterate the need for additional types of prevention methods to be made available. Although reported by women in similar contexts to be a less important attribute of HIV prevention implants (35), our findings are in keeping with existing literature which suggests that there may be some preference for a biodegradable compared to a non-biodegradable implant, an attribute that may be linked to women's perception of method privacy or discretion (21, 36–38, 42, 43).

The differences noted across population groups, and the influence of factors such as sexual behaviour, condom use, and trying to conceive, indicate that preferences are not homogenous, and that women need a choice of prevention methods to suit their different and likely changing preferences and needs.

### Limitations

The following limitations should be considered. Firstly, preferences and product choices were self-reported and hypothetical. Real life decision making may be influenced by social and contextual factors and may differ from what is self-reported. Although the influence of workshop participation was adjusted for in the final models, this may have resulted in an over reporting of the proportion of women who would consider an MPT-implant if they were not provided with sufficient opportunity to discuss new method choices. We acknowledge that participation in the workshop activities allowed those participants to engage more in discussions on product attributes. The activities also differed slightly between workshops, and there may have been a social desirability bias among workshop attendees, or dominant workshop participants who may have influenced the groups' preferences. Participants were predominantly recruited from health facilities, and thus may reflect a population already accessing prevention services, who may differ from the general population. Participants were all English speaking, and the results may therefore not accurately reflect those of individuals who could not speak English. In the statistical analysis, we did not account for the different sampling methods, or the potential associations between participants recruited through the snowball sampling, which may limit the representativeness of the sample. The restriction of this study to pill, injectable and implant products limits the considerations and influence that the availability of a vaginal ring method may have on women's prevention product choices. In addition, the study did not include event-driven and on-demand methods, for which women have shown interest (44).

## Conclusions

In this study exploring women's preferences for prevention methods, women indicated a preference for products which provide dual and long term protection, with few side effects. Their method choices were influenced be sexual-behavioural factors and current and prior contraceptive method use. Providing a prevention method choice and a population specific approach to new method development and introduction could enhance their ability to fill a gap in prevention needs. Ensuring access to accurate, client-centred information around products prior to their introduction may also enhance uptake and use.

## Data Availability

The raw data supporting the conclusions of this article will be made available by the authors, without undue reservation.
