# Supplementary material for: Prevention method preferences and factors influencing hypothetical choice among women in South Africa: a survey exploring opportunities for a multipurpose prevention technology implant
Source: Front Reprod Health. 2024 Jun 25;6:1368889. doi: 10.3389/frph.2024.1368889 (PMC11231390; doi:10.3389/frph.2024.1368889)
Supplement: Supplementary file 1 [file Table1.docx]

Table 1. Demographic and sexual behavioural characteristics of participants, by population group

|  | **AGYW 18 – 24 years**  N = 289 (34.2%) | | **Women >24 years**  N = 374 (44.3%) | | **Female sex workers**  N = 40 (4.7%) | | **Total**  N = 703 (100%) | |
| --- | --- | --- | --- | --- | --- | --- | --- | --- |
| **Age (years)** |  |  |  |  |  |  |  |  |
| Mean (SD) | 20.9 | (2.00) | 30.7 | (4.37) | 31.7 | (6.09) | 26.7 | (6.15) |
| **Study site** |  |  |  |  |  |  |  | |
| KZN | 99 | 34.3% | 149 | 39.8% | 0 | 0.0% | 248 | 35.3% |
| Gauteng | 102 | 35.3% | 104 | 27.8% | 40 | 100.0% | 246 | 35.0% |
| Eastern Cape | 88 | 30.4% | 121 | 32.4% | 0 | 0.0% | 209 | 29.7% |
| **Sexual orientation** |  |  |  |  |  |  |  | |
| Heterosexual | 256 | 88.6% | 358 | 95.7% | 30 | 75.0% | 644 | 91.6% |
| Bisexual/Homosexual | 28 | 9.7% | 13 | 3.5% | 7 | 17.5% | 48 | 6.8% |
| Missing | 5 | 1.7% | 3 | 0.8% | 3 | 7.5% | 11 | 1.6% |
| **Highest level of education completed** | | | | | | | | |
| Primary school or lower | 12 | 4.2% | 10 | 2.7% | 4 | 10.0% | 26 | 3.7% |
| Secondary school | 210 | 72.7% | 215 | 57.5% | 28 | 70.0% | 453 | 64.4% |
| College or University | 60 | 20.8% | 142 | 38.0% | 5 | 12.5% | 207 | 29.4% |
| Missing | 7 | 2.4% | 7 | 1.9% | 3 | 7.5% | 17 | 2.4% |
| **Employment** |  |  |  |  |  |  |  |  |
| Student | 133 | 46.0% | 37 | 9.9% | 2 | 5.0% | 172 | 24.5% |
| Employed (full/part time/self) | 26 | 9.0% | 87 | 23.3% | 20 | 50.0% | 133 | 18.9% |
| Unemployed | 124 | 42.9% | 243 | 65.0% | 17 | 42.5% | 384 | 54.6% |
| Missing | 6 | 2.1% | 7 | 1.9% | 1 | 2.5% | 14 | 2.0% |
| **Ever had sexual intercourse** | 257 | 88.9% | 367 | 98.1% | 40 | 100.0% | 664 | 94.5% |
| **Early sexual debut** | 8 | 2.8% | 10 | 2.7% | 2 | 5.0% | 20 | 2.8% |
| **Ever given birth** | 99 | 34.3% | 307 | 82.1% | 37 | 92.5% | 443 | 63.0% |
| **Relationship** |  |  |  |  |  |  |  |  |
| No sexual partner | 83 | 28.7% | 95 | 25.4% | 10 | 25.0% | 188 | 26.7% |
| Casual partners | 73 | 25.3% | 88 | 23.5% | 11 | 27.5% | 172 | 24.5% |
| Married or in a committed relationship | 120 | 41.5% | 173 | 46.3% | 11 | 27.5% | 304 | 43.2% |
| Other/Unknown | 13 | 4.5% | 18 | 4.8% | 8 | 20.0% | 39 | 5.5% |
| **Knowledge of partner's HIV status**^*^ | | | | |  |  |  |  |
| Known | 133 | 64.6% | 167 | 59.9% | 20 | 66.7% | 320 | 62.1% |
| Unknown | 73 | 35.4% | 112 | 40.1% | 10 | 33.3% | 195 | 37.9% |
| **More than one sexual partner in the last 3 months**^*^ | | | | |  |  |  |  |
|  | 13 | 6.3% | 24 | 8.6% | 15 | 50.0% | 52 | 10.1% |
| **Condom use at last sex**^†^ | 124 | 48.3% | 191 | 52.0% | 24 | 60.0% | 339 | 51.0% |
| **Transactional sex in the last 3 months** | | | | |  |  |  |  |
|  | 24 | 8.3% | 55 | 14.7% | 32 | 80.0% | 111 | 15.8% |
| **Trying to conceive** | 22 | 7.6% | 38 | 10.2% | 4 | 10.0% | 64 | 9.1% |
| **Current contraceptive use**^†^ |  |  |  |  |  |  |  |  |
| None^**^ | 87 | 33.9% | 96 | 26.2% | 10 | 25.0% | 193 | 29.0% |
| Condoms only | 15 | 5.8% | 50 | 13.6% | 7 | 17.5% | 72 | 10.8% |
| Implant | 37 | 14.4% | 40 | 10.9% | 7 | 17.5% | 84 | 12.7% |
| Injectable | 89 | 34.6% | 127 | 34.6% | 7 | 17.5% | 223 | 33.6% |
| IUD | 1 | 0.4% | 4 | 1.1% | 0 | 0.0% | 5 | 0.8% |
| Pill | 20 | 7.8% | 35 | 9.5% | 8 | 20.0% | 63 | 9.5% |
| Female or male sterilization | 3 | 1.2% | 5 | 1.4% | 0 | 0.0% | 8 | 1.2% |
| Unknown | 5 | 2.0% | 10 | 2.7% | 1 | 2.5% | 16 | 2.4% |
| **Used a contraceptive implant before** | 52 | 18.0% | 96 | 25.7% | 13 | 32.5% | 161 | 22.9% |
| **Ever tested for HIV** | 268 | 92.7% | 355 | 94.9% | 38 | 95.0% | 661 | 94.0% |
| **Ever had an STI** | 57 | 19.7% | 105 | 28.1% | 14 | 35.0% | 176 | 25.0% |
| **Ever used PrEP before** | 118 | 40.8% | 73 | 19.5% | 34 | 85.0% | 225 | 32.0% |
| **Ever used PEP before** | 41 | 14.2% | 63 | 16.8% | 12 | 30.0% | 116 | 16.5% |
| **Some perceived risk of HIV** | 61 | 21.1% | 124 | 33.2% | 17 | 42.5% | 202 | 28.7% |
| **Some perceived risk of STI** | 92 | 31.8% | 166 | 44.4% | 22 | 55.0% | 280 | 39.8% |
| **Some perceived risk of pregnancy** | 84 | 29.1% | 142 | 38.0% | 15 | 37.5% | 241 | 34.3% |

^*^ Among those with a partner (n=515)

† Among those who had ever had sex (n=664)

^**^ Includes those who reported not using contraceptives or using only abstinence, emergency contraception or traditional contraceptive methods (e.g., withdrawal)
