# Supplementary material for: Prevention method preferences and factors influencing hypothetical choice among women in South Africa: a survey exploring opportunities for a multipurpose prevention technology implant
Source: Front Reprod Health. 2024 Jun 25;6:1368889. doi: 10.3389/frph.2024.1368889 (PMC11231390; doi:10.3389/frph.2024.1368889)
Supplement: Supplementary file 2 [file Table2.docx]

Table 2: Hypothetical prevention product choices among participants, by population group

|  | **AGYW 18 – 24 years**  N = 289 (41.1%) | | **Women >24 years**  N = 374 (53.2%) | | **Female sex workers**  N = 40 (5.7%) | | **Total**  N = 703 (100%) | | **P-value** |
| --- | --- | --- | --- | --- | --- | --- | --- | --- | --- |
| ***Most important factor in choice of prevention product*** | | | | | | | | | 0.016 |
| Provides dual protection | 65 | 22.5% | 65 | 17.4% | 12 | 30.0% | 142 | 20.2% |  |
| Side effects are manageable | 33 | 11.4% | 93 | 24.9% | 7 | 17.5% | 133 | 18.9% |  |
| Offers long term protection | 39 | 13.5% | 42 | 11.2% | 5 | 12.5% | 86 | 12.2% |  |
| Size of product | 16 | 5.5% | 35 | 9.4% | 1 | 2.5% | 52 | 7.4% |  |
| Convenient to use | 19 | 6.6% | 20 | 5.3% | 4 | 10.0% | 43 | 6.1% |  |
| Available for use | 17 | 5.9% | 17 | 4.5% | 1 | 2.5% | 35 | 5.0% |  |
| Dissolvable | 12 | 4.2% | 18 | 4.8% | 3 | 7.5% | 33 | 4.7% |  |
| Flexible/suits my lifestyle | 20 | 6.9% | 9 | 2.4% | 2 | 5.0% | 31 | 4.4% |  |
| Administration by a provider required | 13 | 4.5% | 13 | 3.5% | 1 | 2.5% | 27 | 3.8% |  |
| Effectiveness | 11 | 3.8% | 12 | 3.2% | 1 | 2.5% | 24 | 3.4% |  |
| Method of use | 10 | 3.5% | 11 | 2.9% | 1 | 2.5% | 22 | 3.1% |  |
| Low user burden | 6 | 2.1% | 8 | 2.1% | 1 | 2.5% | 15 | 2.1% |  |
| Avoidance of painful removal and scarring | 9 | 3.1% | 4 | 1.1% | 0 | 0.0% | 13 | 1.8% |  |
| Discreet | 7 | 2.4% | 4 | 1.1% | 1 | 2.5% | 12 | 1.7% |  |
| Frequency of dosing | 4 | 1.4% | 3 | 0.8% | 0 | 0.0% | 7 | 1.0% |  |
| Missing | 8 | 2.8% | 20 | 5.3% | 0 | 0.0% | 28 | 4.0% |  |
| ***What you would like to prevent*** |  |  |  |  |  |  |  |  | 0.082 |
| Only one (HIV/STI/pregnancy) | 11 | 3.8% | 33 | 8.8% | 2 | 5.0% | 46 | 6.5% |  |
| HIV & Pregnancy | 61 | 21.1% | 59 | 15.8% | 5 | 12.5% | 125 | 17.8% |  |
| HIV & STI | 14 | 4.8% | 26 | 7.0% | 2 | 5.0% | 42 | 6.0% |  |
| HIV, pregnancy & STI | 202 | 69.9% | 251 | 67.1% | 30 | 75.0% | 483 | 68.7% |  |
| Unknown | 1 | 0.3% | 5 | 1.3% | 1 | 2.5% | 7 | 1.0% |  |
| ***Would consider using for the prevention of HIV*** | | | | | | | | |  |
| Once daily pill | 100 | 34.6% | 173 | 46.3% | 23 | 57.5% | 296 | 42.1% | 0.001 |
| Monthly pill | 178 | 61.6% | 244 | 65.2% | 30 | 75.0% | 452 | 64.3% | 0.216 |
| Two monthly injectable | 137 | 47.4% | 199 | 53.2% | 21 | 52.5% | 357 | 50.8% | 0.325 |
| Six monthly injectable | 138 | 47.8% | 204 | 54.5% | 20 | 50.0% | 362 | 51.5% | 0.218 |
| ***Would consider using for the prevention of HIV and pregnancy*** | | | | | | | | | |
| 1-year MPT non-biodegradable implant | 142 | 49.1% | 227 | 60.7% | 27 | 67.5% | 396 | 56.3% | 0.004 |
| 1-year MPT biodegradable implant | 144 | 49.8% | 184 | 49.2% | 23 | 57.5% | 351 | 49.9% | 0.607 |
| 1-year refillable MPT implant | 103 | 35.6% | 182 | 48.7% | 27 | 67.5% | 312 | 44.4% | <0.001 |
| 2-year non-biodegradable MPT implant | 126 | 43.6% | 205 | 54.8% | 17 | 42.5% | 348 | 49.5% | 0.011 |
| 2-year biodegradable MPT implant | 144 | 49.8% | 176 | 47.1% | 22 | 55.0% | 342 | 48.6% | 0.553 |
| ***Would consider any 1- or 2-year MPT implant*** | | | | | | | | | |
|  | 246 | 85.1% | 333 | 89.0% | 38 | 95.0% | 617 | 87.8% | 0.111 |
| ***Choice if only one prevention method available*** | | | | | | | | | 0.001 |
| Once daily pill | 26 | 9.0% | 19 | 5.1% | 1 | 2.5% | 46 | 6.5% |  |
| Monthly pill | 55 | 19.0% | 53 | 14.2% | 3 | 7.5% | 111 | 15.8% |  |
| Two monthly injectable | 19 | 6.6% | 36 | 9.6% | 1 | 2.5% | 56 | 8.0% |  |
| Six monthly injectable | 46 | 15.9% | 75 | 20.1% | 2 | 5.0% | 123 | 17.5% |  |
| 1-year MPT non-biodegradable implant | 16 | 5.5% | 40 | 10.7% | 2 | 5.0% | 58 | 8.3% |  |
| 1-year MPT biodegradable implant | 28 | 9.7% | 37 | 9.9% | 9 | 22.5% | 74 | 10.5% |  |
| 1-year refillable MPT implant | 16 | 5.5% | 21 | 5.6% | 7 | 17.5% | 44 | 6.3% |  |
| 2-year non-biodegradable MPT implant | 30 | 10.4% | 32 | 8.6% | 6 | 15.0% | 68 | 9.7% |  |
| 2-year biodegradable MPT implant | 46 | 15.9% | 53 | 14.2% | 9 | 22.5% | 108 | 15.4% |  |
| None | 6 | 2.1% | 3 | 0.8% | 0 | 0.0% | 9 | 1.3% |  |
| Missing | 1 | 0.3% | 5 | 1.3% | 0 | 0.0% | 6 | 0.9% |  |
